# People living with HIV on ART have accurate perception of lipodystrophy signs: a cross-sectional study

**DOI:** 10.1186/s13104-017-2377-3

**Published:** 2017-01-13

**Authors:** Paulo R. Alencastro, Nemora T. Barcellos, Fernando H. Wolff, Maria Letícia R. Ikeda, Fabiana Schuelter-Trevisol, Ajácio B. M. Brandão, Sandra C. Fuchs

**Affiliations:** 1 Hospital Sanatório Partenon, State Department of Health, Rio Grande do Sul. Av. Bento Gonçalves, 3722, Porto Alegre, RS 90650-001 Brazil; 2 Post-graduate Program of Collective Health, Universidade do Vale do Rio dos Sinos, Av. Unisinos, 950, São Leopoldo, RS 93022-750 Brazil; 3 Postgraduate Studies Program in Sciences in Gastroenterology and Hepatology, School of Medicine, Universidade Federal do Rio Grande do Sul, R. Ramiro Barcelos 2600, Porto Alegre, RS 90035-003 Brazil; 4 Postgraduate Studies Program in Cardiology, School of Medicine, Universidade Federal do Rio Grande do Sul, R. Ramiro Barcelos 2600, Porto Alegre, RS 90035-003 Brazil; 5 Post Graduate Studies Program in Medicine-Hepatology, Universidade Federal de Ciências da Saúde de Porto Alegre, R. Sarmento Leite, 245, Porto Alegre, RS 90050-170 Brazil; 6 Division of Cardiology, Centro de Pesquisa Clínica, 5º andar, Hospital de Clínicas de Porto Alegre, Rua Ramiro Barcellos, 2350, Porto Alegre, RS 90035-903 Brazil

**Keywords:** HIV, AIDS, Lipodystrophy, Lipohypertrophy, Lipoatrophy

## Abstract

**Background:**

The prevalence of lipodystrophy ranges from 31 to 65%, depending on the criteria adopted for diagnosis. The usual methods applied in the diagnosis vary from self-perception, medical examination, skinfolds measurements, or even imaging assessment for confirmation of fat distribution changes. Although several methods have been developed, there is no gold standard for characterization of LA and LH, or mixed forms. This study aimed to compare self-reported signs of lipodystrophy with objective measures by skinfolds and circumferences, and to evaluate the prevalence of lipoatrophy (LA) and lipohypertrophy (LH) among subjects living with HIV/AIDS on ART.

**Methods:**

A cross-sectional study enrolled participants living with HIV/AIDS receiving ART, aged 18 years or older from an outpatient health care center, in Southern Brazil. Self-reported body fat enlargement in the abdomen, chest or breasts, and dorsocervical fat pad were used to determine LH, while LA was identified by self-reported fat wasting of the face, neck, legs, arms or buttocks. Measurements were obtained with a scientific caliper for infraorbital, buccal, and submandibular skinfolds, and using an inelastic tape to measure circumferences of waist, hip, neck, and arm. LH and LA were established by the presence of at least one self-reported sign.

**Results:**

Comparisons of self-reported signs with objective measurements for men and women were carried out in 815 participants on ART, out of 1240 participants with HIV infection. Self-report of decreased facial fat and sunken cheeks was associated with lower infraorbital, buccal, and submandibular skinfolds. Participants who reported buffalo hump had, on average, greater neck circumference, as well as those who have increased waist circumference also reported abdominal enlargement, but no buttock wasting. Men were most commonly affected by lipoatrophy (73 vs. 53%; P < 0.001), and women by lipohypertrophy (79 vs. 56%; P < 0.001).

**Conclusion:**

In conclusion, self-reported signs of lipodystrophy and lipoatrophy are prevalent, differ by gender, and are associated with objective measurements in people living with HIV/AIDS.

## Background

Highly active antiretroviral therapy (HAART) has increased the survival and quality of life of people living with HIV/AIDS. However, the use of antiretroviral therapy (ART) is associated with metabolic abnormalities, including increased serum lipids, glucose, and insulin resistance. Combined, these disorders represent an atherogenic profile, increasing the risk of developing cardiovascular disease [1]. The interaction of host factors, HIV, and HAART leads to the accumulation and loss of body fat in specific body sites [2], which has been identified as lipodystrophy [3]. Lipodystrophy includes peripheral subcutaneous fat loss (lipoatrophy; LA) in the upper and lower limbs, buttocks, and face as well as increased body fat, (lipohypertrophy; LH) detected as abdominal visceral fat accumulation, dorsocervical fat pad (buffalo hump), and chest or breast enlargement. LA and LH can be presented separately or in combination in the same participant (mixed forms) [4].

The prevalence of lipodystrophy ranges from 31 to 65% [5–11] depending on the criteria adopted for diagnosis. The usual methods applied in the diagnosis vary from self-perception [6, 12], health professional [12] or physical examination [7], skinfolds measurements [8], or even imaging assessment such as dual emission X-ray absorptiometry (DEXA) [7, 9], CT [9], or MRI [10] for confirmation of fat distribution changes [10, 11]. Although several methods have been developed, there is no gold standard for characterization of LA and LH, or mixed forms [2, 13]. A multicenter case–control study was designed to develop a sensitive, specific, and broadly applicable definition of lipodystrophy for people living with HIV, providing a model with a reduced number of variables. The model included clinical data as age, duration of HIV infection, HIV disease clinical stage, change in CD4+ count from nadir, and waist circumference [9]. This model was improved by a neural network analysis with an input of a large set of variables. The neural network model was 72% accuracy (72% sensitivity and 71% specificity), compared with 68% (73% sensitivity and 63% specificity) of the first one [14]. However, the models have not been used in clinical practice. One of them requires expensive imaging exams, as well as large number of variables [14] and they were not incorporated in the routine diagnosis of lipodystrophy.

The diagnostic approach most commonly used to detect lipodystrophy is self-report of specific changes in body fat distribution [15, 16]. Physicians detect LA or LH signs during clinical examination [6], but changes in body fat depends on repeated observations though continuous care of patients by the same doctor [15]. A few studies require both patient and doctor agreement on signs of lipodystrophy [15, 17]. Few scales have attempted to quantify the intensity of body fat alterations to compute an overall score [17–19]. However, the comparison between self-reported signs and objective measurements through skinfolds and circumferences is lacking. Therefore, this study aimed to compare self-reported signs of lipodystrophy: LA and LH with objective measurements of skinfolds and circumferences. In addition, this study also aimed to compare prevalence of LA and LH between men and women living with HIV on ARV.

## Methods

This cross-sectional study enrolled a consecutive sample of men and women living with HIV/AIDS on ART, from June 2006 to December 2008. Participants aged 18 years or older, who consecutively sought HIV diagnostic confirmation or treatment at the HIV/AIDS outpatient care center in Southern Brazil and were invited to participate. Pregnant women, intellectually impaired participants, and incarcerated or institutionalized persons were excluded. The outpatient care center—Hospital Sanatorio Partenon—is one of three larger centers, which provide AIDS Care and Treatment for patients living in any area of the metropolitan area.

### Study variables

A standardized questionnaire was used to collect data on demographic and socioeconomic characteristics [20], and another questionnaire to obtain data on use of antiretroviral drugs and signs of lipodystrophy [21] among other variables. Race was self-reported and categorized as Caucasian or non-Caucasian. Education was determined as the number of years at school, as a socioeconomic status proxy. Highly active antiretroviral therapy was defined as the use of three or more drugs over the 12 months preceding the interview. Body changes were assessed, both subjectively (by report) and objectively (by examination). Changes in body fat were classified as reduced, increased or none.

Lipodystrophy was defined as the self-perception of changes in body fat distribution through questions about changes in specific regions, such as face (hollow cheeks, double chin); neck (enlargement and/or buffalo hump); chest (or breasts) and abdomen enlargement; arms, forearms, hands, thighs, legs and feet (muscular arms and legs, prominent superficial veins); hips and/or buttocks wasting. The question about presence of double chin was used to capture the perception of increased fat in the participant’s face and for internal consistency as well. The questions about neck enlargement and buffalo hump aimed to detect the accumulation of fat in the dorsocervical spine, but they represent the same sign.

Lipohypertrophy was assessed by the accumulation of fat in the abdomen, chest or breasts, and dorsocervical region. Lipoatrophy was determined by the fat wasting of face, neck, legs, arms, and buttocks. Lipohypertrophy and lipoatrophy were established by the presence of at least one self-reported sign [11, 21] of hypertrophy or atrophy. Mixed lipodystrophy was diagnosed by the simultaneous presence of atrophic and hypertrophic changes [22]. Objective measurements of skinfolds were measured with a scientific calipers in the infraorbital, buccal, and submandibular regions, and using an inelastic tape to measure waist, hip, neck, and arm circumferences.

### Data collection

Physicians and research assistants conducted standardized interviews, but only physicians measured blood pressure and anthropometric parameters. Approximately 5% of interviews were repeated, by an independent researcher, for quality control purpose. Participants underwent laboratory testing (blood chemistry, viral load measurement, and CD4 counts), using standard techniques. Weight (kg) and height (m) were measured, with participants in barefoot and wearing light clothes, and body mass index (BMI) was calculated by dividing weight (kg) by height (in m^2^). Waist circumference was measured midway between **t**he uppermost border of the iliac crest and the lower border of the costal margin (rib cage) [23]. Hip circumference was measured at the greater trochanter and the point of greatest gluteal protuberance. Neck circumference was measured 2 cm below the cricoid cartilage, while arm circumference was assessed at the midpoint between the acromion and olecranon. A flexible inelastic tape was employed to perform circumference measurements in triplicate. Facial skinfolds were measured with scientific skinfold calipers in the infraorbital, buccal, and submandibular regions [24], by a physician who attended a session on AIDS Clinical Trials Group: Anthropometric Measurement Training, in which the protocol used in this study was standardized. Before the start of the study, we confirmed the reproducibility in a few participants. Skinfold thicknesses were measured in duplicate. The average was calculated for all measurements carried out in duplicate or triplicate.

### Calculation of sample size and statistical analysis

To detect a prevalence of lipohypertrophy between 6 and 8%, with a 2% error and a confidence interval of 95%, the required sample size varied from 445 to 772 participants on ART. A sample of 778–1000 participants would be sufficient to detect a 9% difference in the prevalence of lipodystrophy between men and women, with a P value of 0.05, 80% statistical power, and a male-to-female ratio of 1:1. A total of 1240 participants were investigated and 815 on ART were analyzed. The sample size was calculated using Epi Info™ version 3.4.1 (US Centers for Disease Control and Prevention, Atlanta, GA).

The prevalence of lipodystrophy between men and women was analyzed using Pearson’s Chi squared test or analysis of variance (ANOVA) for comparison of means. Among the self-reported characteristics of body fat distribution, we selected those that could be used to characterize lipodystrophy, lipoatrophy and lipohypertrophy through their association with objective measurements of the corresponding body regions. All analyses were performed in the Statistical Package for the Social Sciences (SPSS^®^) version 16 (Chicago, IL, United States).

## Results

Among 1295 HIV-infected subjects, 1240 were enrolled, 15 refused to take part, and 40 were excluded due to pregnancy, intellectual disability for signing a consent form, be incarcerated or institutionalized. Approximately a third of the participants were ARV naïve, therefore, the analysis was focused on 815 ART participants. As Table 1 shows, men were older, had higher number of years at school, were predominantly Caucasians, and had lived longer with HIV/AIDS. Women had higher body mass index, but there was no gender difference in CD4 cell counts and undetectable viral load. Approximately 65% of women versus half of men were on protease inhibitor (PI), but no association with gender was observed for thymidine analogue nucleoside reverse transcriptase inhibitors (tNRTI) (P = 0.9).

**Table 1:** Characteristics of people living with HIV on ART, stratified by gender [N (%) or mean ± SD]

	Overall (N = 815)	Men (N = 628)	Women (N = 612)	P value
Age (years)	40.0 ± 9.7	41.5 ± 9.7	38.4 ± 10.1	<0.001
Caucasian ethnicity	457 (56.1)	380 (60.5)	312 (51.0)	<0.001
Years of formal education	7.4 ± 4.1	8.0 ± 4.2	6.8 ± 3.8	<0.001
Body mass index (kg/m^2^)	24.6 ± 4.3	24.0 ± 3.5	25.3 ± 5.0	<0.001
Time since HIV diagnosis (years)	6.0 ± 4.2	6.3 ± 4.5	5.7 ± 3.9	0.03
CD4 (cells per μL)				0.3
>350	451 (55.6)	221 (53.0)	230 (58.4)	
200–350	215 (26.5)	116 (27.8)	99 (25.1)	
<200	145 (17.9)	80 (19.2)	65 (16.5)	
Viral load <50 copies/mL	488 (60.3)	261 (62.7)	227 (57.8)	0.15
Protease inhibitors use (PI)	468 (37.7)	212 (50.5)	256 (64.8)	<0.001
Thymidine analogue nucleoside reverse transcriptase inhibitors (tNRTI)	253 (31.0)	131 (31.2)	122 (30.9)	0.9
Duration of PI (≥5 years)	105 (12.9)	61 (14.5)	44 (11.1)	0.15
Duration of tNRTI (≥4.5 years)	255 (31.3)	145 (34.5)	110 (27.8)	0.04

**Fig. 1:**
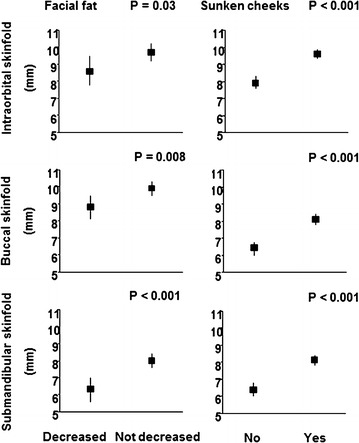
Face: self-perception changes vs. skinfold measurements (mean ± SD)

Figure 1 describes the means and standard deviations of skinfolds measures according to self-perceived changes of decreased body fat distribution in the face. Self-reported signs of LA were associated with decreased infraorbital, buccal, and submandibular skinfolds.

**Fig. 2:**
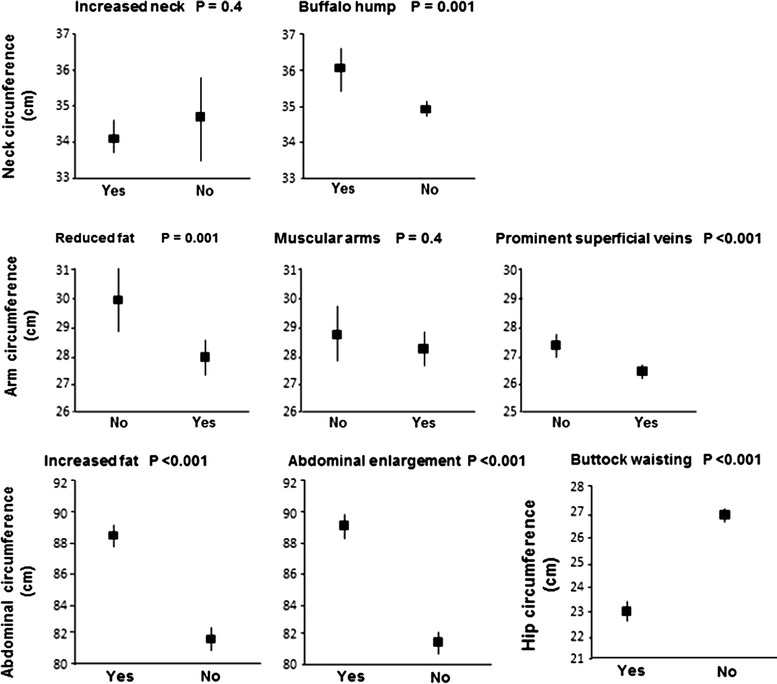
Fat body distribution: self-reported changes vs. circumference measurements (mean ± SD)

Figure 2 shows means and standard deviations of circumference measurements for several body areas. Self-reported signs of LH were associated with increased neck, arm, waist and hip circumferences. Participants who reported buffalo hump had an average of neck circumference higher than those who did not report. A reduced arm circumference was associated with the self-report of superficial prominent veins. There was a strong association between increased waist circumference and self-reported signs of increased fat and abdominal enlargement; while buttock wasting was inversely associated with the average hip circumference.

**Fig. 3:**
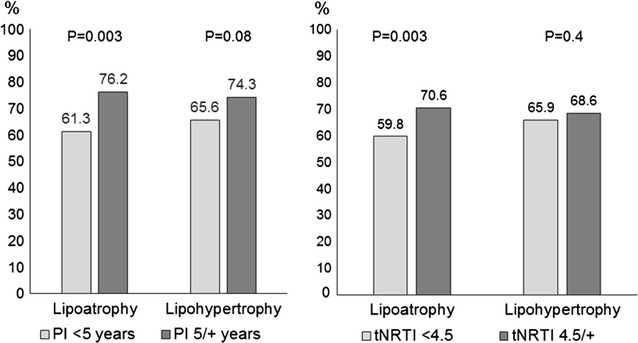
Prevalence of lipoatrophy and lipohypertrophy by use of PI and tNRTI

Table 2 shows the prevalence of self-reported changes in body fat distribution for lipoatrophy and lipohypertrophy in men and women. There were significant differences for all self-perceived signs, including the number of reported signs. Men had higher prevalence of fat facial wasting, hollow cheeks, arm wasting, and prominent veins in the arms. Approximately two-thirds of women have reported abdominal enlargement or abdominal fat. It is noteworthy that 31% of men reported three or more lipoatrophy signs, making it difficult not notice. On the other side, only 12% of women had three signs of LH. Most participants on ART have LA or LH, with the former being more prevalent among men and the latter in women. Figure 3 shows that lipoatrophy was highly prevalent among those on tNRTI, but there was no association between PI and lipohypertrophy.

**Table 2:** Prevalence of lipoatrophy and lipohypertrophy signs according to self-perception (%) by gender among participants on ART

	Overall (N = 815)	Men (N = 420)	Women (N = 395)	P value
Reported signs of lipoatrophy				
Fat facial wasting	32.6	38.3	26.6	<0.001
Hollow cheeks	31.2	36.9	25.1	<0.001
Buttock wasting	30.9	33.8	27.8	0.07
Arm wasting	24.5	30.7	18.0	<0.001
Prominent veins in the arms	30.3	41.7	18.2	<0.001
Number of reported signs of lipoatrophy				<0.001
0	36.8	26.9	47.3	
1	23.1	24.0	22.0	
2	14.2	17.6	10.6	
≥3	25.9	31.4	20.0	
Reported signs of lipohypertrophy				
Buffalo hump	10.6	6.2	15.2	<0.001
Increased abdominal fat	57.8	48.3	67.8	<0.001
Abdominal enlargement	53.3	40.5	66.8	<0.001
Number of reported signs of lipohypertrophy				<0.001
0	33.3	44.5	21.3	
1	19.1	19.0	19.2	
2	40.4	33.3	47.8	
3	7.2	3.1	11.6	
Prevalence of at least one reported sign				
Lipoatrophy	515 (63.2)	307 (73.1)	208 (52.7)	<0.001
Lipohypertrophy	544 (66.7)	233 (55.5)	311 (78.7)	<0.001

## Discussion

This study identified self-reported signs of LA and LH in 815 participants on ART that were significantly associated with objective measures used to evaluate the fat redistribution. LA signs of facial fat wasting and hollow cheeks were associated with lower infraorbital, buccal and submandibular skinfold. On the other hand, LH signs were associated with increased circumferences such as abdomen enlargement detected by increased waist circumference and buffalo hump by larger neck circumference. Prevalence of lipoatrophy was more prevalent in men and lipohypertrophy in women. Although the differences between men and women do not constitute formal validation test, comparative analysis of self-reported signs of changes in body fat distribution with specific measurements supports the use of the former in clinical practice.

The use of skinfold or circumference measurements is more objective than clinical observation, but is limited by the absence of cutoffs for facial skinfolds, for instance [22]. Circumference cutoff points, which have been defined for other conditions, also require specific validation as predictors of HIV-associated lipodystrophy. The prevalence of lipodystrophy in this study was greater than that detected in a dynamic cohort of ambulatory patients from seven cities in the US [18]. In the US study, 4% of participants had moderate to severe signs of LH and 18% LA signs. In this study, prevalence rates were higher than those observed in a cross-sectional study conducted in São Paulo, Brazil, where 37% of participants had LH, 49% LA, and 22% mixed lipodystrophy [25]. Although our and that study of São Paulo used the same criteria to classify changes in fat distribution, in the latter at about 10% of participants used tNRTI for more than 36 months versus one-third using it for 4.5 years or more in our study. In this sense, the present study showed different manifestations of LA and LH when comparing men and women. Similarly to previous studies, our results suggest that increase in fat distribution in regions of the body were slightly more frequent in the women than men [22, 25]. In addition, our results were confirmatory for the differences between men and women in the prevalence of LA and LH. The finding that lipoatrophy was more prevalent among men [26] and lipohypertrophy among women [27, 28] has been observed in other studies that used different fat distribution assessments. Our study also confirmed previous results showing higher prevalence of lipoatrophy among participants who have used tNRTI [29].

This study identified high rates of self-reported signs of lipodystrophy, which were significantly associated with objective measurements. The number of self-reported signs and objective measures used to establish abnormality used herein was arbitrary, but was more conservative than those adopted in previous studies. The number of self-reported signs differed significantly between men and women.

Among the study limitations is the inability to compare self-report signs with a reference standard for skin folds and circumferences. The establishment of cutoffs for skinfold and circumferences would make easier to identify LA and LH. However, this is the case of clinical practice in resource-limited settings [30]. In developing countries, access to quantification of regional fat with DEXA, computed tomography, or magnetic resonance imaging [7, 31]. is limited and does not cover assessment of lipodystrophy. The implementation of standardized criteria for the diagnosis of LA and LH in people living with HIV can provide relevant data to detect this condition and to compare results across studies.

## Conclusion

In conclusion, self-reported signs of lipodystrophy and lipoatrophy are prevalent, differ by gender, and are associated with objective measurements among HIV-infected adults receiving ART. Therefore, the use of subjective allows the detection of lipoatrophy and lipohypertrophy, but objective measures allow their quantification.

## Abbreviations

LA: lipoatrophy
LH: lipohypertrophy
HAART: highly active antiretroviral therapy
ART: antiretroviral therapy
DEXA: dual emission X-ray absorptiometry
BMI: body mass index
ANOVA: analysis of variance
SPSS^®^: Statistical Package for the Social Sciences
PI: protease inhibitor
tNRTI: thymidine analogue nucleoside reverse transcriptase inhibitors
